# Supplementary material for: Combination of a Bioceramic Scaffold and Simvastatin Nanoparticles as a Synthetic Alternative to Autologous Bone Grafting
Source: Int J Mol Sci. 2018 Dec 18;19(12):4099. doi: 10.3390/ijms19124099 (PMC6321089; doi:10.3390/ijms19124099)
Supplement: Supplementary file 1 [file ijms-19-04099-s001.pdf]

International Journal of
Molecular Sciences

Supporting information

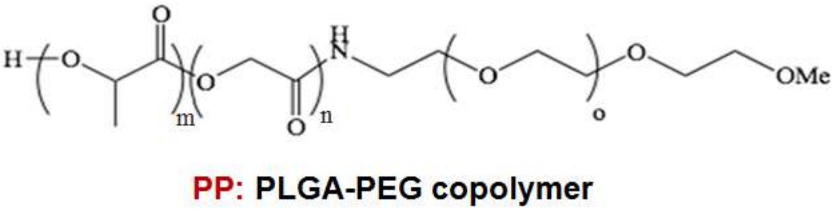

(**a**)

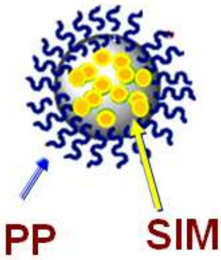

(b1)

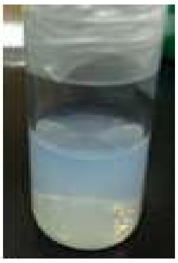

(b2)

Figure S1. The methoxy-functionalized diblock copolymer of PLGA-PEG-methoxy (PP) was synthesized by conjugating the heterofunctional PEG with a terminal amine and carboxylic acid functional group to PLGA-COOH using standard *N*,*N*′-dicyclohexylcarbodiimide (DCC)-mediated chemistry (**a**). The simvastatin (SIM) agent was encapsulated in PLGA-PEG-methoxy nanoparticles to form SIM-PP NPs by the precipitation-solvent evaporation technique (**b1**,**b2**).

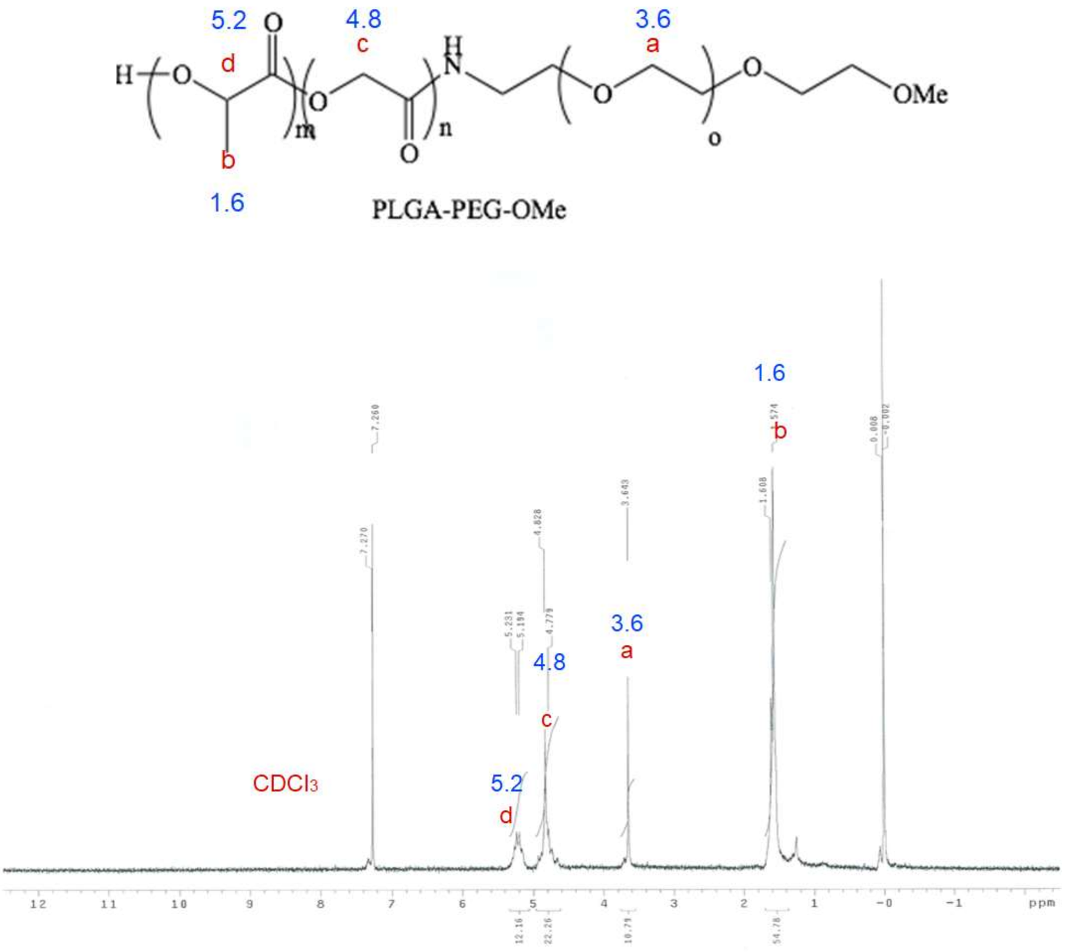

Figure S2. ^{1}H NMR spectrum confirms to the PLGA-PEG-OMe structure. ^{1}H NMR ($CDCl_3$, 200 MHz) d: δ5.219(m, 11H), c: δ4.667-4.817(m, 22H), b: δ3.641(s,1H), a: δ1.562(m,33H).

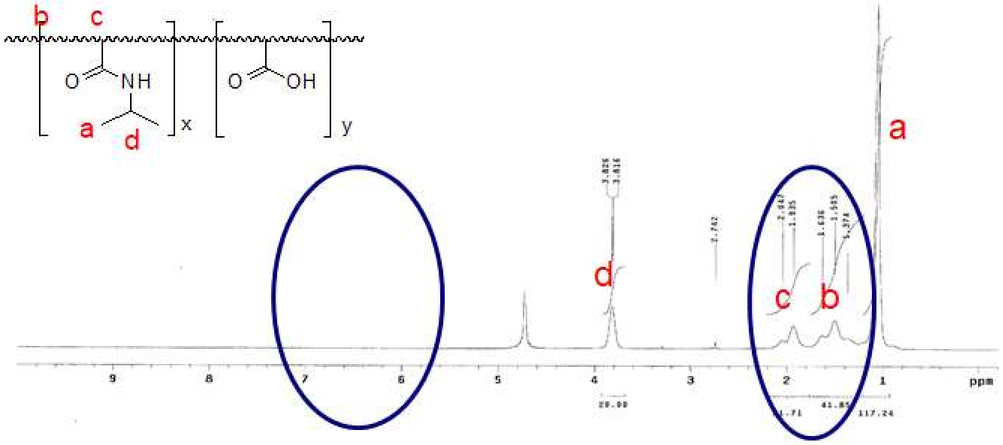

Figure S3. H spectrum of p(NiPAAm-co-MAA) = 97:3 (PNM97:3) copolymer were examined by NMR.

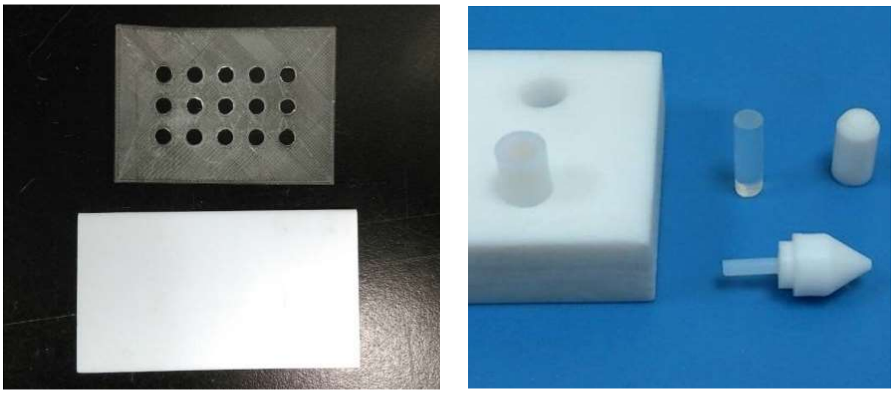

(**a1**) (a2)

Figure S4. Two types of plastic molds for forming biphasic (hydroxyapatite and β-calcium phosphates) porous bone substitutes: a disk mold (**a1**, ϕ5 mm; h 0.7 mm) and hollow cylinder mold (**a2**, ϕ_1 3.5 mm; ϕ_2 1.5 mm; h 10 mm).

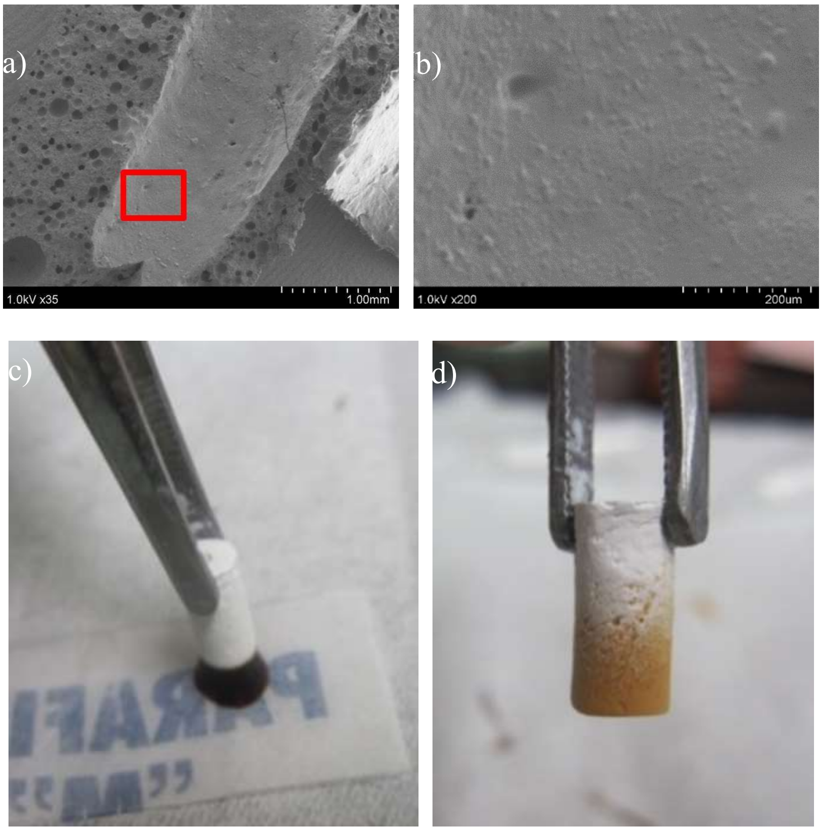

Figure S5. The SEM morphology of the SIM-PP NPs loaded within porous bioceramic of hollow cylinder after sintering at 1200 °C for 2h (**a**,**b**). The porous bioceramic of hollow cylinder absorb red ink to rise photos by liquid permeability test (**c**,**d**).

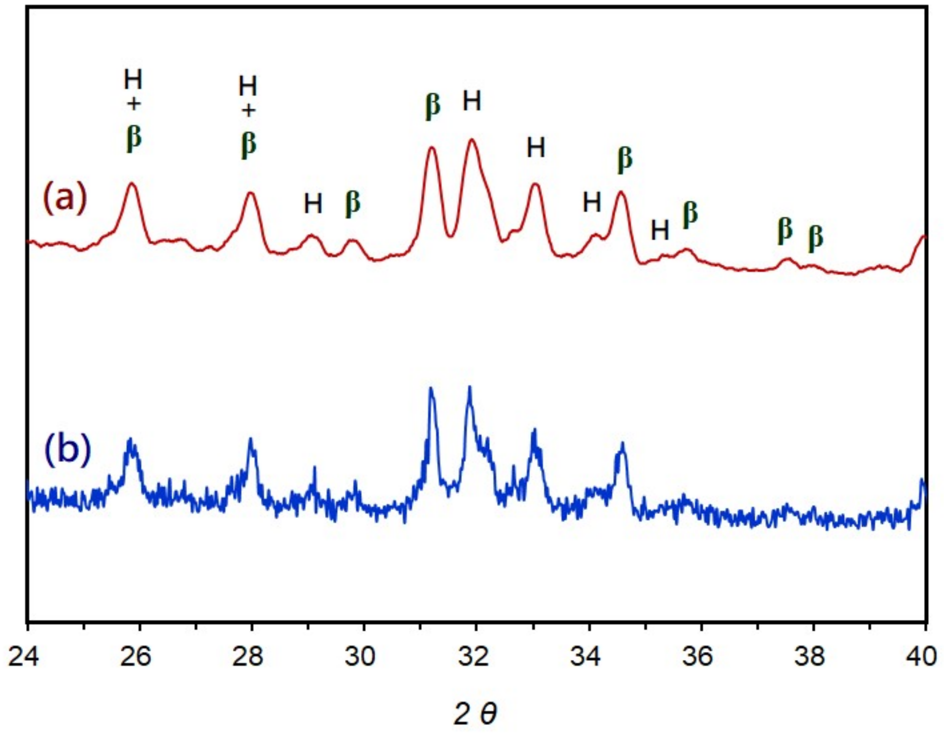

Figure S6. XRD patterns of the porous HAp/β-TCP (50/50) bioceramics after 1200 °C sintered for 2 h (**a**), commercial raw materials of HAp/β-TCP (**b**). (β = β-TCP, H = HAp).

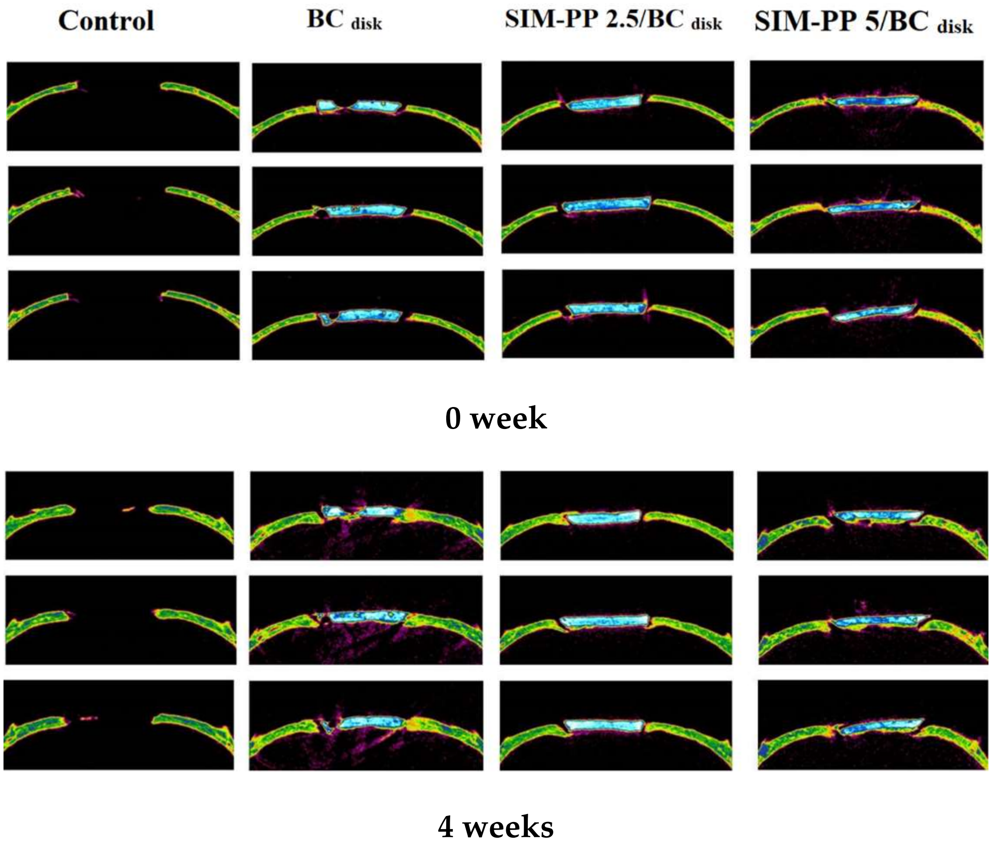

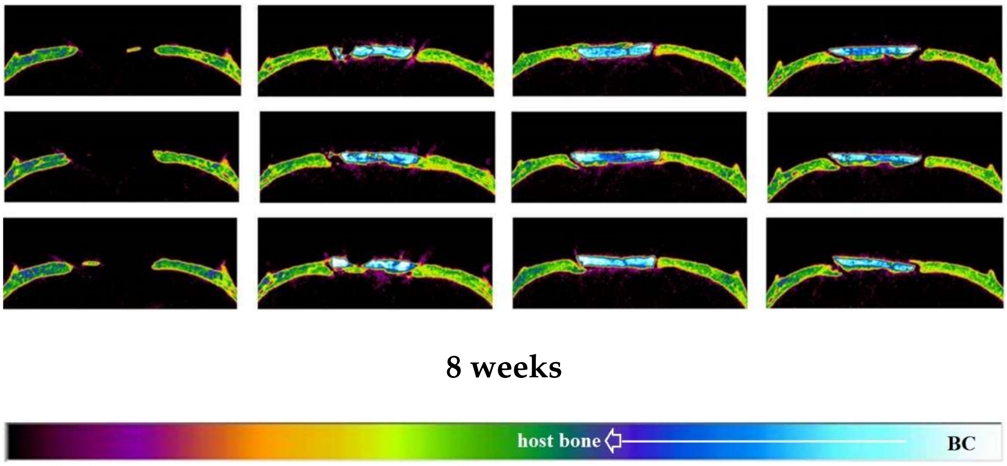

Figure S7. Cross-section slices of calvarial bone from the Hounsfield unit [HU] calibration of micro-CT images for the rat model at 8 weeks after implantation of BC_{disk} samples (ϕ5 mm; h 0.7 mm), 2.5 μmol of SIM in SIM-PP/BC_{disk} samples and 5.0 μmol of SIM in SIM-PP/BC_{disk} samples. Notes: calvarial bone defects only were used as controls and the rat number is tree).
